# MICALL2 as a substrate of ubiquitinase TRIM21 regulates tumorigenesis of colorectal cancer

**DOI:** 10.1186/s12964-022-00984-3

**Published:** 2022-10-28

**Authors:** Pushuai Wen, Huade Wang, Yi Li, Xinyao Sui, Zhijuan Hou, Xiaoyan Guo, Wanying Xue, Dahua Liu, Yu Wang, Jing Gao

**Affiliations:** 1 Department of Ultrasonography, The First Affiliated Hospital, Jinzhou Medical University, No. 2, Section 5, Renmin Street, Jinzhou, 121001 China; 2 Department of Pathophysiology, Jinzhou Medical University, No.40, Section 3, Songpo Road, Jinzhou, 121001 China; 3 Biological Anthropology Institute, Jinzhou Medical University, No.40, Section 3, Songpo Road, Jinzhou, 121001 China; 4 Life Science Institute, Jinzhou Medical University, No.40, Section 3, Songpo Road, Jinzhou, 121001 China; 5 Department of Hematology-Oncology, Fuyang Sixth People’s Hospital, Fuyang, 236000 China

**Keywords:** Colorectal cancer, MICALL2, TRIM21, Ubiquitination, Wnt signaling pathway

## Abstract

**Background:**

Molecule interacting with CasL-like protein 2 (MICALL2) is believed to regulate cytoskeleton dynamics, tight junction formation, and neurite outgrowth. However, its biological role and the underlying mechanism in colorectal cancer (CRC) remain largely elusive.

**Methods:**

qRT-PCR, Western blotting and immunohistochemistry assays were used to detect the expression levels of different genes. Next, mass spectrometry, co-immunoprecipitation and immunofluorescence staining were used to detect the interactions of proteins. Furthermore, MTT assay, colony formation assay, wound-healing assays and xenograft tumor models were performed to demonstrate the functions of MICALL2 in CRC. In addition, transcriptome sequencing and Western blotting were conducted to verify the mechanism of MICALL2 in CRC.

**Results:**

We found that both mRNA and protein levels of MICALL2 are up-regulated in colorectal cancer tissues compared with non-tumor tissues and that its overexpression is closely correlated with poor prognosis. Ubiquitin E3 ligase Tripartite motif-containing protein 21 (TRIM21) mediated MICALL2 ubiquitination and proteasome-dependent degradation, negatively correlated with MICALL2 levels, and reversely regulated the tumorigenic activity of MICALL2 in CRC. Functional studies confirmed that MICALL2 promoted colorectal cancer cell growth and migration via the Wnt/β-catenin signaling pathway.

**Conclusions:**

As a substrate of ubiquitinase TRIM21, MICALL2 enhances the growth and migration of colorectal cancer cells and activates the Wnt/β-catenin signaling pathway.

**Video abstract**

**Supplementary Information:**

The online version contains supplementary material available at 10.1186/s12964-022-00984-3.

## Background

Colorectal cancer (CRC) is the third most frequent type of cancer in the world and accounts for the second highest number of cancer-related deaths [1]. The mechanism of colorectal cancer development remains incompletely understood, which has become the main bottleneck for CRC prevention and treatment. Therefore, further research into the regulatory mechanisms of colorectal cancer tumorigenesis could lead to more effective diagnosis, prognosis, and targeted therapy.

The MICAL (molecule interacting with CasL) family is a multi-structural domain protein that is expressed in specific neuronal and non-neuronal cells during development and adulthood, and consists of a flavin-adenine dinucleotide binding domain (FAD), calponin homology (CH), Lin11, Isl-1 and Mec-3 (LIM) and bivalent Mical/EHBP Rab binding (bMERB) domain structural domains [2]. A unique structural feature of MICAL is its FAD domain, which can produce H_2_O_2_ and oxidatively modifies methionine on actin to depolymerize actin [3]. Currently, the majority of research focused on the function of MICAL family that regulates cytoskeletal dynamics [3–7]. Functionally, MICAL regulate Drosophila bristle formation, dendritic spine construction, axon guidance, and hippocampal mossy fiber attachment [7–10]. However, MICALL2 has distinct activities, like as endocytosis and tight junction formation, due to the absence of the FAD structural domain [11–13]. Furthermore, depending on the cellular context, several MICAL family members function either as tumor suppressors or oncogenes and are implicated in the cancer cell growth, migration, invasion and angiogenesis [14–17]. However, the biological function of MICALL2, its role in cancer signal transduction, and its clinical significance in human colorectal cancer are still remained elusive.

Protein post-transcriptional modification (PTM), such as phosphorylation, ubiquitination, acetylation, methylation, and glycosylation, is one of the most influential mechanisms for adaption to rapid changes in the internal and external signals [18]. Mical phosphorylation by Abl non-receptor tyrosine kinase directly enhances its redox-mediated F-actin disassembly [19]. However, other PTMs of MICAL have not been well addressed in colorectal cancer to date. TRIM21, a RING finger domain- containing E3 ligase, is a member of the tripartite motif (TRIM) family. TRIM21 is made up of N-terminal RING domain with E3 ubiquitin ligase activity, B-box domain, coiled-coil domain, PRY and SPRY domains at the C-terminal [20]. As an ubiquitin E3 ligase, TRIM21 could mediate the ubiquitination and degradation of many interacting proteins, including TRPM2, IRF-8, SQSTM1/p62, Oct-1, Par-4, p21, p53, c-FLIP and BCL2, and may be involved in regulation of the inflammation, immune response, cell metabolism, redox homeostasis, and cancer [21–27].

In this study, we first reported that E3 ubiquitin-protein ligase TRIM21 interacted with and ubiquitinated MICALL2 to modulate its stability via the proteasome-dependent pathway. And, we also discovered the potential oncogenic role of MICALL2 on colorectal cancer cell tumorigenicity by activating the Wnt/β-catenin signaling cascade. Our findings shed light on the biological role of MICALL2 in the process of colorectal cancer and suggest that MICALL2 could be a potential target for colorectal cancer therapy.

## Materials and methods

### Human colorectal cancer tissues

A total of 27 cases of colorectal cancer tissues and matching adjacent non-tumor colorectal tissues were collected from the First Affiliated Hospital of Jinzhou Medical University, China (No. 201923). And, all patients enrolled in the current study provided written informed consent. All sample collection and processing were undertaken according to the Declaration of Helsinki and ethical approval was obtained from the Ethics Committee of the First Affiliated Hospital of Jinzhou Medical University.

### Cell culture

The human colorectal carcinoma cell lines (HCT116, HCT8, SW480) and HEK293 were cultured in the medium with 10% FBS (Biological Industries, Kibbutz Beit Haemek, Israel), penicillin (100 unit/mL), and streptomycin (100 μg/mL) in a 37 °C humidified atmosphere of 5% CO_2_.

### Antibodies and reagents

The antibodies against MICALL2, TRIM21, Lamin B1, β-catenin, E-cadherin, Vimentin, c-Myc, Cyclin D1, Flag tag, His tag, Ubiquitin, and GAPDH were purchased from Proteintech (Proteintech, Chicago, IL, USA). The Lipofectamine 3000 and TRIzol™ reagent were purchased from Invitrogen (Invitrogen, Carlsbad, CA, USA). Nuclear and cytoplasmic extraction kit was purchased from Thermo Fisher Scientific (Waltham, MA, USA). The PAGE Gel Silver Staining kit (Cat. No. G7210) and MG132 (IM0310) were supplied by Beijing Solarbio Science & Technology Co., Ltd. (Beijing, China). Cycloheximide (CHX) (Cat. No.A49960) was purchased from Shanghai Acmec Biochemical Co., Ltd. (Shanghai, China).

### Plasmids construction

The plasmids of pcDNA3.1-His-MICALL2, p3xFlag-CMV-TRIM21 and other truncated mutants were constructed based on human cDNAs of MICALL2 and TRIM21 were purchased from Miaolingbio Bioscience & Technology Co., Ltd. (Wuhan, China). Two MICALL2 specific targeting shRNAs with the following target sites were cloned in the lentiretroviral vector pLKO.1‐puro (Addgene, Cambridge, MA, USA): shMICALL2#1, 5’- CCGGTCTTGCACACGAGCAGAACTTCTCGAGAAGTTCTGCTCGTGTGCAAGATTTTTG-3’ and 5’-AATTCAAAAATCTTGCACACGAGCAGAACTTCTCGAGAAGTTCTGCTCGTGTGCAAGA-3’; shMICALL2#2, 5’-CCGGTGTCGTCCTTGTAGTACACTTC TCGAGAAGTGTACTACAAGGACGACATTTTTG-3’ and 5’-AATTCAAAAATGTCGTCCTTGTAGTACACTTCTCGAGAAGTGTACTACAAGGACGACA-3’; and shCtr, 5’-CCGGACGTGACACGTTCGGAGAATTCTCGAGAATTCTCCGAACGTGTCACGTTTTTTG-3’ and 5’-AATTCAAAA AACGTGACACGTTCGGAGAATTCTCGAGAAT TCTCCGAACGTGTCACGT-3’.

### Construction of colorectal cancer cell lines with stable overexpression or knockdown of MICALL2

The constructed plasmid with pcDNA3.1-His-MICALL2 or control vector were transfected into HCT116 cells using Lipofectamine^®^ 3000 reagent according to the manufacturer's instruction. After 48 h post-transfection, cells were selected in Geneticin (Invitrogen, Waltham, MA, USA) for 4 weeks, and cell colonies were selected and amplified for further studies. For stable silenced cell lines, HEK293T cells were cotransfected with shMICALL2‐pLKO.1 with packing plasmids by using Lipofectamine^®^ 3000. After 48 h post-transfection, the harvested supernatants containing packaged lentivirus were used to infect HCT8 cells. Puromycin was then added into the culture to screen for stable cell lines.

### Quantitative real-time polymerase chain reaction (qRT-PCR)

Total RNA was extracted from frozen tissue using TRIzol reagent and reverse transcribed into cDNA using a cDNA synthesis kit (Abm, Milton, ON, Canada). Real-time PCR analysis was performed with ABI 7500 real-time PCR system to specifically assess the relative abundances of MICALL2 mRNAs. The MICALL2 and 18S rRNA primers were as follows: MICALL2, 5’-AGTGACATCGTGGACTCGCT-3’ and 5’- TGGAGGCCCAGCTTCTCAATC-3’; 18S rRNA, 5’- GAAACGGCTACCACATCC-3’ and 5’-ACCAGACTTGCCCTCCA-3’. The experiment was repeated three times, and the relative expression level of the target gene was normalized to the mean of 18S rRNA. The data were analyzed using the comparative threshold cycle (2^−ΔΔCT^) method.

### Tissue microarray (TMA) and immunohistochemical (IHC) analyses

Human colorectal cancer TMA (Shanghai Superchip Biotechnology Co., Ltd., Shanghai, China) consisting of 75 colorectal cancer samples and paired adjacent tissue samples were used to perform IHC by the streptavidin-peroxidase method (ZSGB-BIO, Beijing, China). To quantify the expression in the colorectal cancer tissues and matched adjacent non-tumor colorectal tissues, at least five random fields at 200 × magnification in each section were selected. For each field, integrated optical density (IOD) of protein was assessed using Image J software (NIH, Bethesda, MD, USA). Average optical density (AOD = IOD/Area) was used in this study for statistical analysis. Finally, the correlation between the expression levels of protein (AOD) and clinicopathological features in colorectal cancer patients was assessed. Besides, IHC staining scores were obtained to assess the correlation of MICALL2 with TRIM21 expression in serial colorectal cancer tissues by multiplying scores representing the staining intensity and positive cells percentage as described previously [17].

### Immunoprecipitation (IP) and immunoblot analysis

Cell extracts were prepared with cell lysate (50 mM NaF, 1 mM Na_3_VO_4_, 1 mM DTT, 1 mM PMSF, protease inhibitors cocktail). After centrifugation, equal amounts of lysates were used to be immunoprecipitated with antibodies overnight at 4 °C and Protein A/G-Agarose. Subsequently, the immune complexe was subjected to SDS-PAGE. Proteins were transferred to nitrocellulose membranes and probed with the corresponding primary and HRP- conjugated secondary antibodies, as well as chemiluminescence visualization.

### Mass spectrometry analysis

Similar to the IP assay, cellular protein extracts were incubated with MICALL2 antibody followed by protein A/G agarose beads. The recovered protein in the immune complexes associated with MICALL2 or IgG were seperated by gel electrophoresis. The bands that specifically bind to MICALL2 were excised, and liquid chromatography tandem mass spectrometry (LC–MS/MS) was performed at PTM Biolab Hangzhou (Hangzhou, China).

### Nuclear and cytoplasmic fractionation

The cells were harvested after centrifugation and washed twice with PBS. Subsequently, the nuclear and cytoplasmic extracts were prepared in accordance with the NE-PER Nuclear and Cytoplasmic Extraction Kit instruction (Thermo Scientific, Waltham, MA, USA). The purities of the nuclear and cytoplasmic extracts were assessed by Western blot with Lamin B1 and GAPDH antibody, respectively.

### Wound healing assay

Colorectal cancer cells were cultured to confluence on 12-well plates. A wound area was scraped carefully with a 20 µL sterile pipette tip and detached cells were removed. At least five images were taken under an inverted microscope at 0 and 24 h following the wound formation. The wound area was measured using Image-J software (NIH, Bethesda, MD, USA). The cell migration rate (% wound closure) was calculated as follows: cell migration rate = [(wound area at 0 h)—(wound area at 24 h)] / (wound area at 0 h) × 100%.

### Soft agar colony formation assay

Cells were suspended in 0.7% agarose and placed in 12-well plates. Then, the cells were cultured for two weeks, and then colonies were fixed with 4% paraformaldehyde and stained with 1% crystal violet. The number of colonies was counted with Image J software.

### Immunofluorescence (IF) staining

Briefly, cells were cultivated on glassbottom culture dishes and fixed with 4% paraformaldehyde before being rinsed three times with PBS and treated with blocking solution. Then, cells were incubated sequentially with primary antibodies overnight at 4 °C, Alexa Fluor 488 and 594-conjugated secondary antibodies (1:500, Thermo Fisher) for 1 h at 37 °C, and 4,6-diamidino-2-phenylindole (DAPI) nuclear dye for 10 min at room temperature. The cells were imaged using a Leica confocal laser-scanning microscope.

### RNA-Seq and bioinformatics

Total RNA was isolated from MICALL2-overexpressed HCT116 cells and control cells using Trizol Reagent (Thermo Fisher Scientific, Carlsbad, CA, USA), and subsequently purified using the Qiagen MinElute column kit (Germantown, MD, USA). Then, the RNA quality, library construction, and paired-end mRNA next-generation sequencing were sequentially performed on Illumina platform by Novogene Corporation Inc (Beijing, China). Next, the differentially expressed genes (DEGs) were statistically assessed by R/Bioconductor package DESeq2 (version 1.30.1) with the following criteria: |log2 (FoldChange)|> 1 and *P*-adj. < 0.05. Gene Ontology (GO) analysis was performed using the National Institutes of Health Database for Annotation, Visualization, and Integrated Discovery (DAVID, https://david.ncifcrf.gov/home.jsp) public online tool and visualized with R/Bioconductor package Goplot (version 1.0.2). UALCAN datasets was assessed for MICALL2 expression (http://ualcan.path.uab.edu/cgi-bin/ ualcan-res.pl). The GEPIA Database (http://gepia.cancer- pku.cn/ index.html) was used to analyze colorectal cancer patient survival and correlation of different genes expressions.

### Animal studies

The animal experiment was carried out in accordance with a procedure approved by Jinzhou Medical University's Animal Experimentation Ethics Committee (Registration No.2021011501). Briefly, 5 × 10^6^ of MICALL2-overexpressed HCT116 cells and control cells were subcutaneously injected into the right flank of 6 week old female BALB/c athymic nude mice (Charles River, Beijing, China). Tumor size was monitored over a 3-week period using calipers. Tumor volume was estimated according to the following formula: volume = (length × width^2^)/2. Tumors were removed and weighed when the mice were euthanized 21 days following treatment.

### Statistical analysis

GraphPad Prism 8.0 was used for statistical analysis. The Student’s *t*-test or one way analysis of variance (ANOVA) was performed to evaluate the differences between two groups or more than two groups. The chi-square test was performed to analyze the correlation between MICALL2 expression and clinicopathological features. The Pearson’s correlation analysis was performed to analyze the relationship between MICALL2 and TRIM21 expression in the colorectal cancer tissues. The value of *P* < 0.05 was considered statistically significant. The data was presented as mean ± SEM. The following asterisks represent statistical significance: *, *P* < 0.05; **, *P* < 0.01; ***, *P* < 0.001.

## Results

### MICALL2 is up-regulated in human CRC tissues

**Fig. 1:**
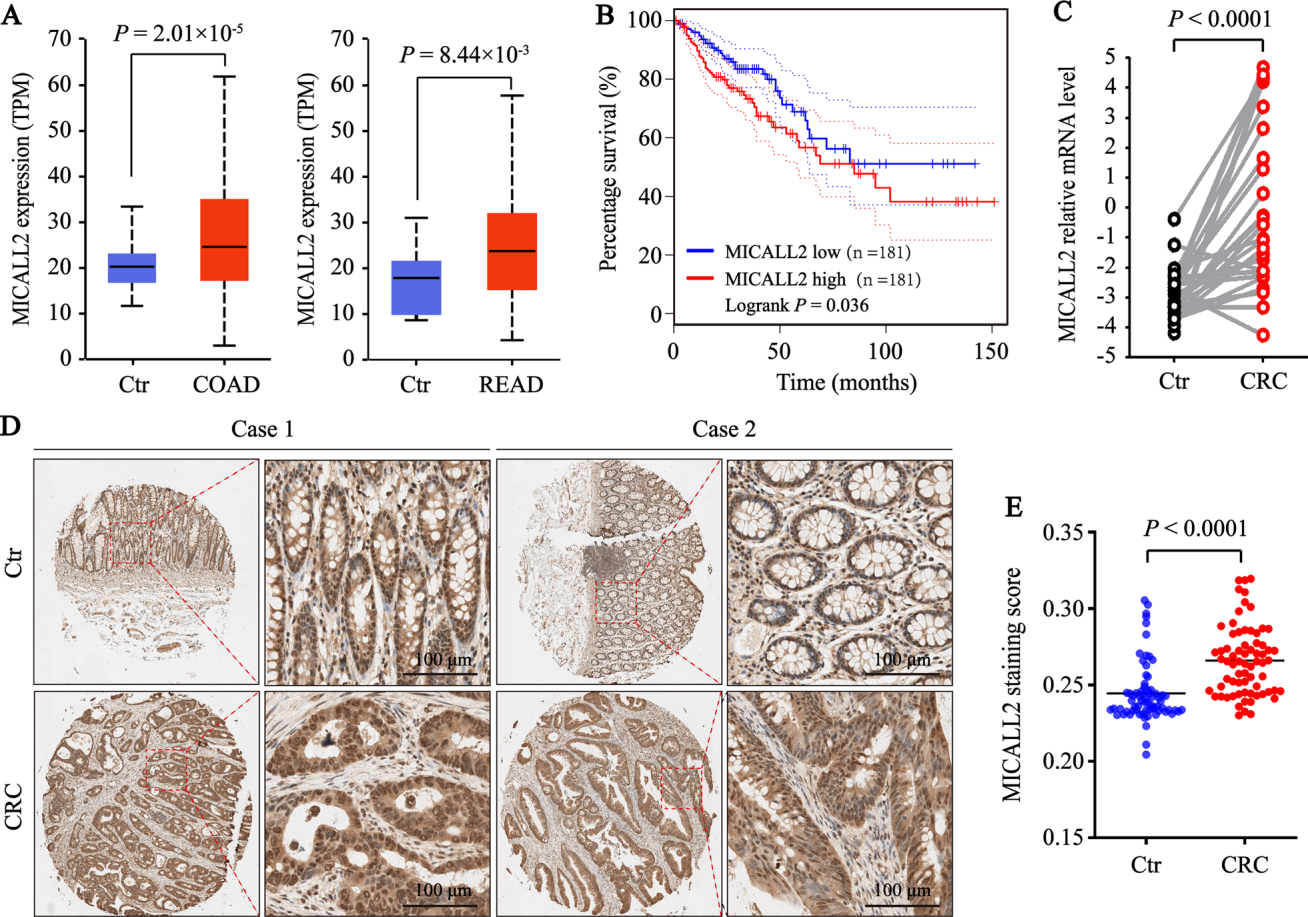
MICALL2 upregulation correlates with poor prognosis in human colorectal cancer. **A** MICALL2 mRNA expression levels in colon adenocarcinoma (COAD) or rectum adenocarcinoma (READ) tissues and normal controls from a TCGA cohort (http://ualcan.path.uab.edu/cgi-bin/ualcan-res.pl). **B** Kaplan–Meier survival analysis of colon carcinoma patients with either lower (blue) or higher (red) MICALL2 expression derived from TCGA data set (http://gepia.cancer-pku.cn/index.html). **C** MICALL2 mRNA expression level in 27 pairs of CRC and adjacent non-tumor tissues (Ctr) was determined by qRT-PCR. **D** Representative IHC staining of MICALL2 in CRC and adjacent non-cancerous colorectal tissues. The positive staining of MICALL2 showed brown, and the nuclei were stained with hematoxylin. **E** The statistical analysis of MICALL2 expression based on the AOD scores in the CRC (n = 66) and adjacent non-cancerous colorectal tissues (n = 66)

The expression levels of MICALL2 were up-regulated in colorectal cancer tissues (Fig. 1A) by using RNAseq data from The Cancer Genome Atlas (TCGA), and Kaplan–Meier survival curve analysis demonstrated that CRC patients with higher MICALL2 protein expression had poorer overall survival rate (Fig. 1B). In line with these data from public database, the mRNA levels of MICALL2 were also significantly up-regulated in the 27 paired human colorectal cancer tissues compared with adjacent non-tumor tissues (Fig. 1C). Furthermore, IHC analysis revealed that the expressions of MICALL2 in colorectal cancer tissues were significantly increased compared with adjacent non-tumor colorectal tissues (Fig. 1D, E). Additionally, higher staining scores were observed in the patients with lymph nodes metastases, as well as those under the age of 60 (Table 1). Collectively, these results suggest that upregulation of MICALL2 may be involved in CRC progression.

**Table 1:** Clinicopathologic variables and the expression status of MICALL2 in colorectal cancer

Variables		Case number (%)	AOD (Mean ± SEM)	*P*-value
Sex	Male	39(0.5909)	0.2417 ± 0.0064	0.1885
	Female	27 (0.4091)	0.2483 ± 0.0098	
Age	< 60	23 (0.3485)	0.2517 ± 0.0114	0.0282
	≥ 60	43 (0.6515)	0.2405 ± 0.0058	
TNM stage	I + II	29 (0.4394)	0.2446 ± 0.0082	0.9295
	III + IV	37 (0.5606)	0.2442 ± 0.0073	
T stage	T1/T2	11 (0.1667)	0.2426 ± 0.0064	0.7491
	T3/T4	55 (0.8333)	0.2447 ± 0.0064	
N stage	N0/N1	53 (0.4848)	0.2407 ± 0.0048	0.0020
	N2	13 (0.5152)	0.2593 ± 0.0204	
M stage	M0	57 (0.8636)	0.2450 ± 0.0049	0.5309
	M1	9 (0.1364)	0.2405 ± 0.0254	

### MICALL2 promotes the growth and migration of colorectal cancer cell

**Fig. 2:**
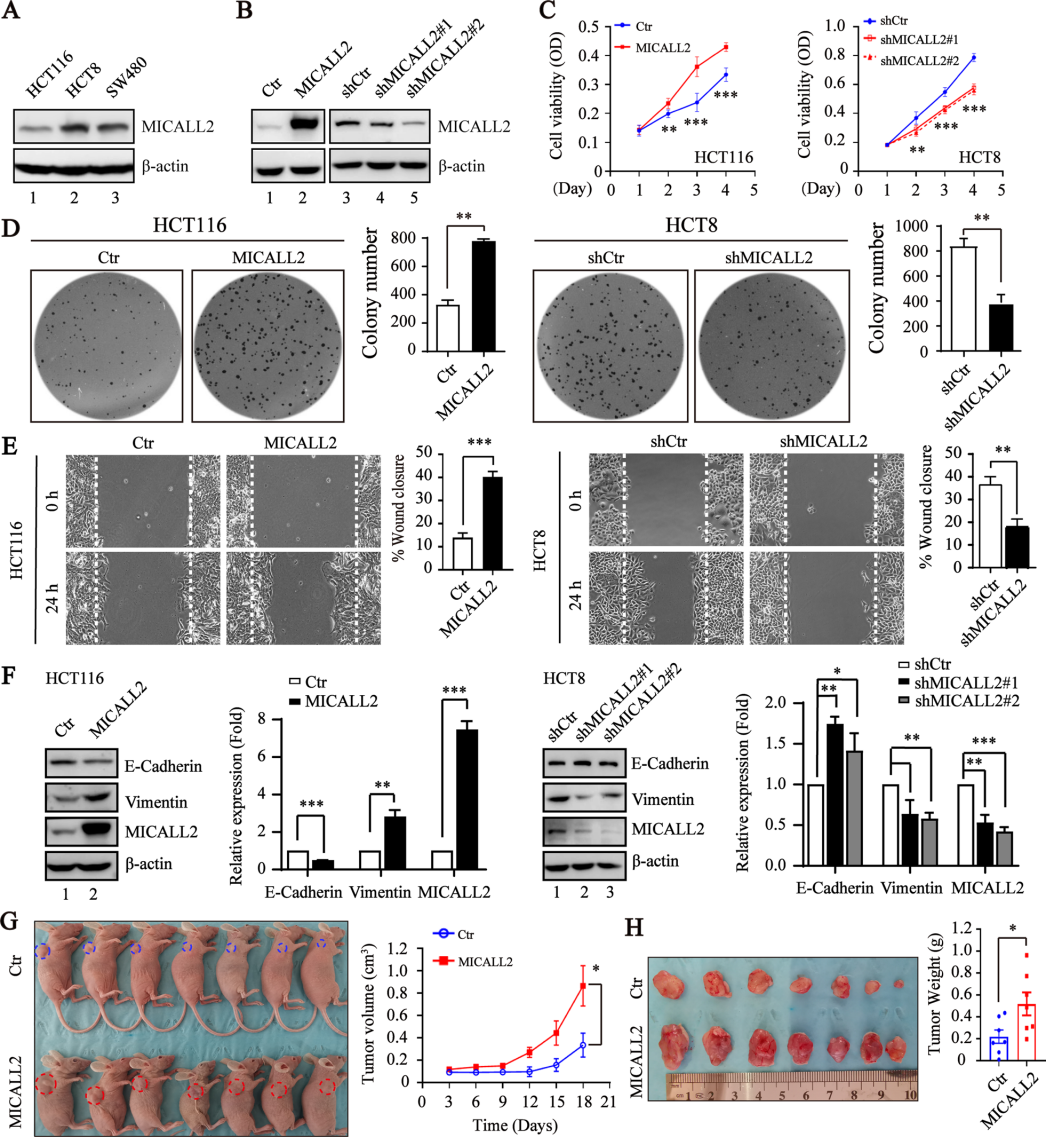
MICALL2 enhances colorectal cancer cell proliferation and migration. **A** The level of endogenous MICALL2 expression in HCT116, HCT8, and SW480 cell lines was detected by Western blotting assay. β-actin was used as a loading control. **B** The expression level of MICALL2 in stable HCT116 cell lines with MICALL2 overexpression and HCT8 cell lines with MICALL2 knockdown was detected by Western blotting assay. β-actin was used as a loading control. **C** MTT growth curves of stable HCT116 cell lines with MICALL2-overexpression of or HCT8 cell lines with MICALL2 knockdown. **D** Colony formation of stable HCT116 cell lines with MICALL2-overexpression of or HCT8 cell lines with MICALL2 knockdown. Representative photos of crystal violet–stained colonies (left) and quantifications results (right) of were shown. **E** Representative images and quantification of gap closure in MICALL2-overexpressed HCT116 and MICALL2-silenced HCT8 cell lines, respectively. **F** The expression level of E-cadherin and Vimentin in MICALL2-overexpressed HCT116 and MICALL2-silenced HCT8 cell lines detected by Western blotting assay, respectively. **G** Representative morphological photographs (left) of the athymic nude mice transplanted subcutaneously with HCT116 cell lines stably expressing empty vector (Ctr) or MICALL2 (MICALL2) (n = 7 per group), and dynamic volume of xenograft tumors (right) was monitored at different time points. **H** Photos for tumors isolated from athymic nude mice at 21 days after injection (left) and tumor weights at 21 days after injection were measured (right)

To explore the potential biological functions of MICALL2 in CRC, the expression levels of MICALL2 in several CRC cell lines were examined firstly. MICALL2-overexpressed HCT116 cell line and MICALL2-stable knockdown in HCT8 cell line were established according to their endogenous MICALL2 expression level (Fig. 2A, B). Next, we examined whether MICALL2 had any effect on CRC tumorigenesis using cell functional assays. MICALL2 overexpression significantly promoted the proliferation compared with that of control group, whereas knockdown of MICALL2 had inhibitory role (Fig. 2C). Similar results were observed using colony formation assay, MICALL2 overexpression significantly increased and knockdown of MICALL2 suppressed colony formation (Fig. 2D). Moreover, the wound closure was promoted in MICALL2-overexpressed HCT116 cells, compared with the control cells, conversely, the wound closure was suppressed in the MICALL2-silenced CRC cells (Fig. 2E). When MICALL2 were overexpressed in CRC cells, the protein expression level of E-cadherin, an epithelial-like cell marker, was downregulated, while the protein expression level of vimentin, a mesenchymal-like cell marker, was elevated. Depleted MICALL2 cells showed enhanced E-cadherin expression but decreased vimentin expression (Fig. 2F). Furthermore, tumor xenograft experiments revealed that MICALL2 overexpression promoted the growth of HCT116 tumors (Fig. 2G, H). Collectively, these results suggested that MICALL2 was crucial in the tumorigenesis of colorectal cancer.

### TRIM21 interacts with MICALL2 in CRC

**Fig. 3:**
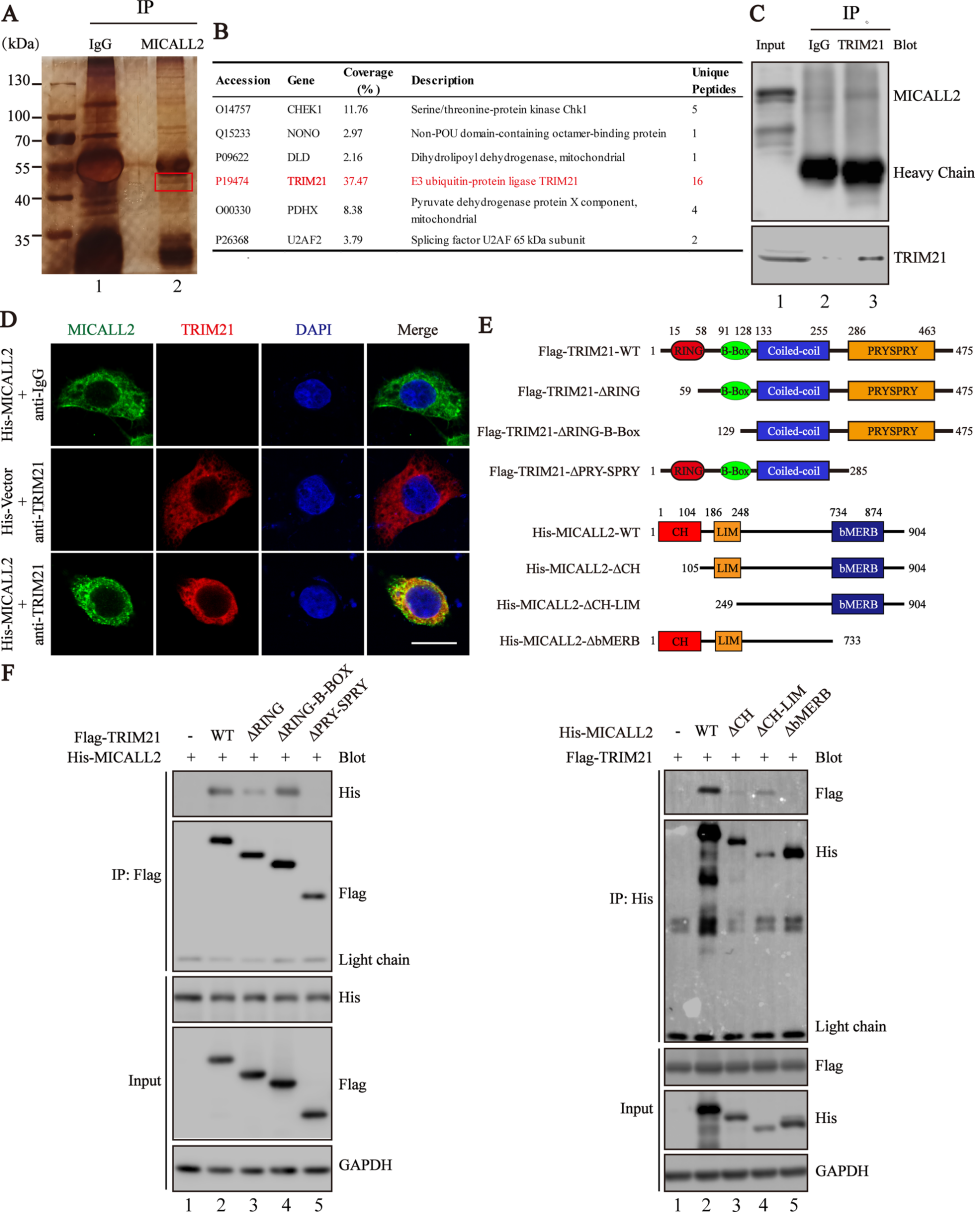
TRIM21 interacts with MICALL2. **A** Silver staining of proteins immunoprecipitated (IP) with MICALL2-antibody with protein extracts from CRC cells followed by identification with LC–MS/MS. The red box indicates one of the most abundant bands as compared with IgG. **B** List of proteins was analyzed by scaffold 4 proteome software and their information is presented. **C** Coimmunoprecipitations were performed to validate the interaction between endogenous MICALL2 and TRIM21 in HCT8 cells. **D** The colocalization of TRIM21 (red) and MICALL2 (green) in HCT8 cells was assessed by laser-scanning confocal microscopy, respectively (scale bar = 10 μm). Nuclei are stained with DAPI (blue). **E** Schematic representation of TRIM21, MICALL2 and its mutants. **F** Co-IP of His-MICALL2 with Flag-tagged TRIM21 and their truncation mutants, or Flag-TRIM21 with His- tagged MICALL2 and their truncation mutants from HEK293 cells. Cells were subjected to immunoprecipitation with α-Flag or His antibody. GAPDH is a housekeeping protein control

The protein pull-down assay and LC/MS–MS analysis were performed to identify candidate proteins that interact with MICALL2. TRIM21, an ubiquitin E3 ligase, was identified as MICALL2 interacting protein (Fig. 3A), and other separated abundant proteins that may physically interact with MICALL2 are also listed in Fig. 3B and Additional file 2: Table S1. Then, the interaction between endogenous MICALL2 and TRIM21 in colorectal cancer cells was validated in the co-immunoprecipitation experiment (Fig. 3C). Furthermore, immunofluorescence staining result revealed that MICALL2 mainly colocalized with TRIM21 in the cytoplasm (Fig. 3D). Different truncated TRIM21 or MICALL2 were constructed to determine which domain mediates its interaction (Fig. 3E). Further domain mapping analysis indicated that the C-terminal PRY-SPRY domain of TRIM21 is required to interact with MICALL2, whereas the bMERB domain is essential for MICALL2 to bind TRIM21 (Fig. 3F).

### TRIM21 mediates the ubiquitination and proteasome-dependent degradation of MICALL2

**Fig. 4:**
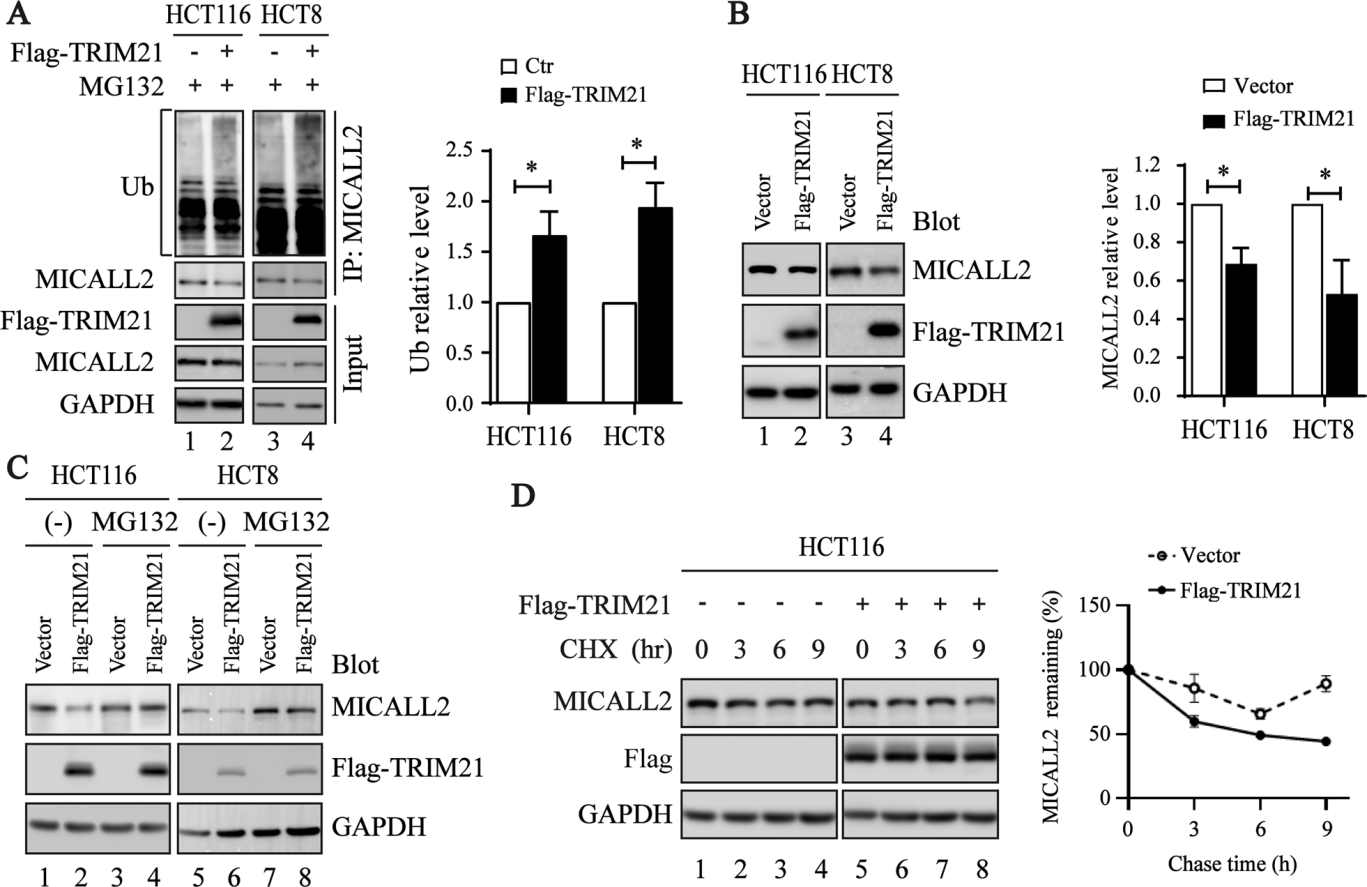
TRIM21 destabilizes MICALL2. **A** The ubiquitination modification (left) and quantification (right) of endogenous MICALL2 was analyzed by immunoprecipitation with anti-MICALL2 antibody and western blotting with anti-ubiquitin antibody in Flag-TRIM21- overexpressed HCT116 or HCT8 and control cells. GAPDH was used as a loading control. **B** Western blotting was used to detect the expression of MICALL2 in the TRIM21-overexpressed HCT116 and HCT8 cell line (left) and the protein levels in different groups were compared to those of GAPDH (right). **C** Western blot analysis of MICALL2 in TRIM21- overexpressed HCT116 and HCT8 cells and control cells treated with MG132 (100 μg/mL) for up to 8 h. **D** Western blot analysis of MICALL2 in HCT116 transfected with plasmids expressing TRIM21 or control plasmid, then treated with CHX (100 μg/mL) for up to 9 h

Given that TRIM21 is an ubiquitin E3 ligase that probably functions through ubiquitylation and subsequent regulation or degradation of target proteins, we evaluate whether TRIM21 is an E3 ligase for MICALL2. As shown in Fig. 4A, overexpression of TRIM21 dramatically elevated the ubiquitination of MICALL2. And, overexpression of TRIM21 can drastically reduce MICALL2 protein levels (Fig. 4B), whereas TRIM21 knockdown increased the MICALL2 protein level (Additional file 1: Figure S1). Moreover, TRIM21 overexpression-mediated degradation of MICALL2 protein could be antagonized by the treatment with MG132, a proteasome-specific inhibitor (Fig. 4C). Finally, as expected, TRIM21 significantly shortened the half-life of MICALL2 (Fig. 4D). Taken together, these results suggest that MICALL2 protein degradation promoted by TRIM21 occurs via the proteasome-dependent pathway.

### TRIM21 negatively correlats with MICALL2 levels and reversely regulates the tumorigenic activity of MICALL2 in CRC

**Fig. 5:**
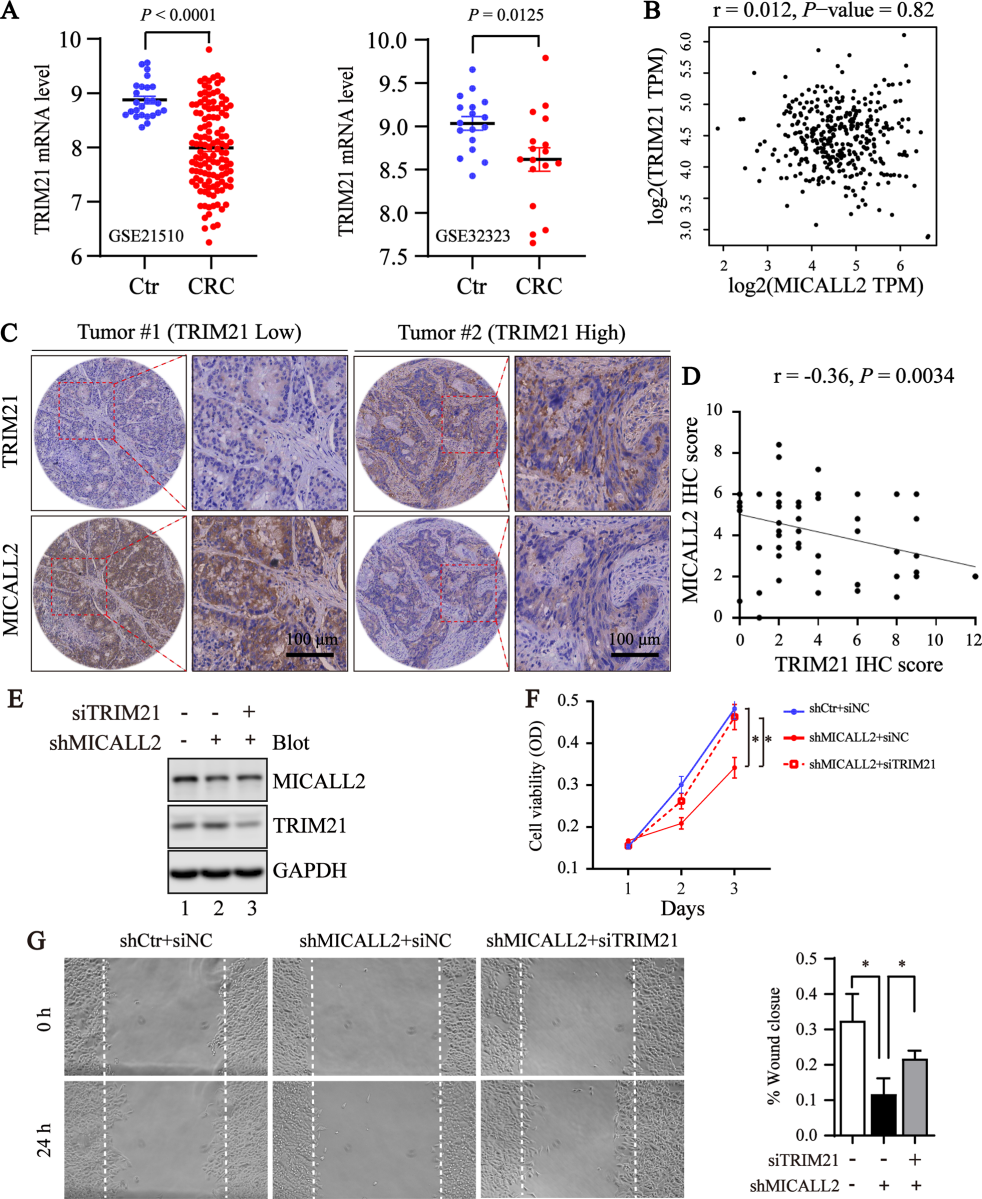
TRIM21 is negatively correlated to MICALL2 and reversely regulates the tumorigenic activity of MICALL2 in CRC. **A** Downregulated TRIM21 mRNA levels in CRC datasets from GEO platform (https://www.ncbi.nlm.nih.gov/geo/). **B** Correlation between TRIM21 and MICALL2 mRNA levels in CRC from TCGA CRC cohort, using GEPIA tool. **C** Representative IHC staining for TRIM21 and MICALL2 in colorectal cancer TMA serial sections. Scale bar are shown as indicated. **D** Negative correlation between TRIM21 and MICALL2 IHC scores in CRC tissues (n = 73). The correlation coefficient (R) and *P* value based on Pearson’s product-moment correlation analysis are shown. **E** Western blotting was performed to detect the expression of MICALL2 and TRIM21 in the stable MICALL2-silenced HCT8 transfected with siRNA targeting TRIM21, and GAPDH was used as loading control. **F** Growth curves of MICALL2-silenced HCT8 cell line transfected with siRNA targeting TRIM21. **G** Representative images (left) and quantification of wound area (right) of gap closure of MICALL2-silenced HCT8 transfected with siRNA targeting TRIM21

The TRIM21 gene expression profile analysis showed that TRIM21 mRNA levels were downregulated in CRC compared with normal tissues (Fig. 5A), which is consistent with the previous study [28]. In addition, there was no discernable relationship between TRIM21 and MICALL2 at the transcriptional level, according to analyses of the TCGA CRC data repository (Fig. 5B), which further suggests that TRIM21 influences the expression of MICALL2 at the post-translational level. Then, we performed IHC to evaluate the potential association between MICALL2 and TRIM21 in the serial sections of human colorectal cancer tissue microarrays (Fig. 5C). Interestingly, the tissues with lower expression of TRIM21 has strong staining of MICALL2, in contrast, the samples with higher expression of TRIM21 displayed low levels of MICALL2 expression. Furthermore, in those colorectal cancer samples, a negative correlation between MICALL2 and TRIM21 proteins was observed (r = − 0.30, *P* < 0.01) (Fig. 5D). To explore the role of TRIM21 in the tumor-promoting function of MICALL2, the effect of TRIM21 knockdown on the tumorigenic activities of MICALL2-stable knockdown HCT8 cell line was examined. The results indicated that MICALL2 depletion-dampened migration and proliferation of CRCs are partially restored by TRIM21 knockdown (Fig. 5E–G). Collectively, TRIM21 reversely regulates the tumorigenic activity of MICALL2 in CRC.

### MICALL2 activates the Wnt/β-catenin signaling pathway in CRC cells

**Fig. 6:**
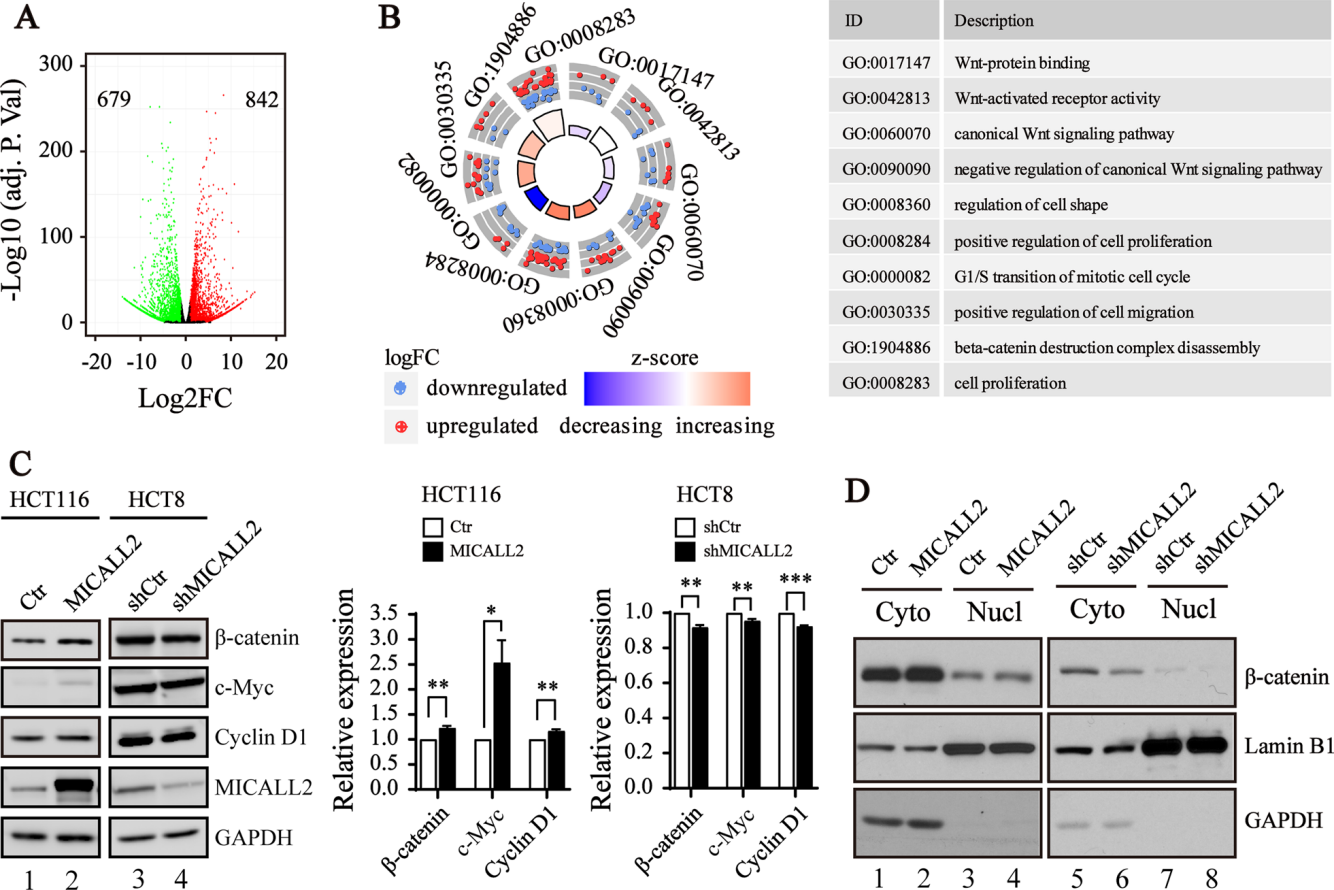
MICALL2 targets Wnt/β-catenin signaling pathways. **A** Volcano plot analysis of transcript expression by RNA-seq of HCT116 cells stably infected with empty vector or MICALL2 expression plasmid. Transcripts in red were significantly upregulated, while those in green were significantly downregulated (logFoldChange > 1, adjust *P* < 0.05). **B** GO enrichment circle plots for DEGs between the HCT116 cells stably infected with MICALL2 expression plasmid or empty vector (left). The outer circle depicts the relative fold change, with blue and red dots representing characteristics that are down-regulated and up-regulated, respectively. The inner quadrants are colored based on the z-score and their surface is a function of the enrichment *P*-value. The associated tables present the GO term ID and function (right). **C** Western blotting was used to assess the expression of β-catenin, C-myc and Cyclin D1 in the MICALL2-overexpressed HCT116 cell line or MICALL2-silenced HCT8 (left) and the protein levels in different groups were compared to that of GAPDH (right). **D** The β-catenin levels in cytoplasmic and nucleic fraction of MICALL2-overexpressed HCT116 cell and or MICALL2-silenced HCT8 lysates were analyzed by Western Blot (left), and the β-catenin levels in cytoplasmic and nucleic fraction were normalized to GAPDH or Lamin B1, respectively. Cyto, cytoplasm; Nucl, nuclear

To study the potential mechanisms that MICALL2 regulates tumorigenesis in CRC, we performed genomewide RNA-Seq in MICALL2 overexpressing and control HCT116 lines. As indicated in Fig. 6A, a total of 1521 differentially expressed genes (DEGs) were identified, including 679 down-regulated and 842 up-regulated genes (Fig. 6A). Go analysis of DEGs revealed that “regulation of cell shape”, “positive regulation of cell proliferation”, “G1/S transition of mitotic cell cycle” and several Wnt signaling pathways were most significantly affected by MICALL2 (Fig. 6B). These data imply that MICALL2 exerts its effects through activating the Wnt/β-catenin signaling pathway, which plays important roles in growth and migration of CRC cells [29]. Notably, MICALL2 overexpression increased the protein levels of β-catenin, and its downstream target molecule (c-Myc and cyclin D1) in HCT116 cells. In HCT8 cells, Knockdown of MICALL2 showed the opposite effect (Fig. 6C). Furthermore, the level of β-catenin in nucleic fraction was increased significantly in MICALL2-overexpressed cells, whereas MICALL2 knockdown caused the opposite effect (Fig. 6D). These results provide convincing evidence that MICALL2 activates the Wnt/β-catenin signaling pathway in CRC.

### Overexpression of β-catenin reverses the effect of MICALL2 knockdown on CRC.

**Fig. 7:**
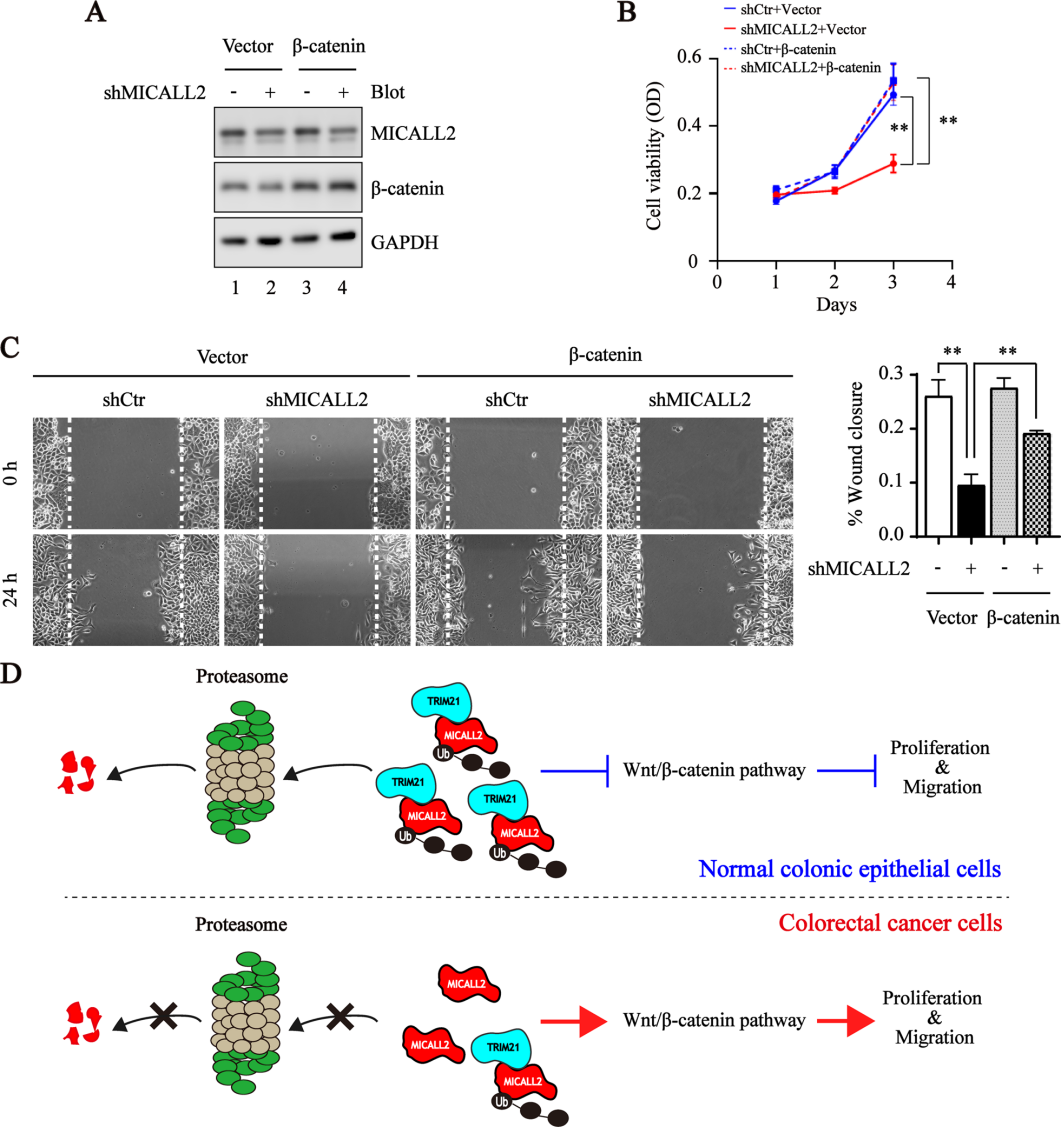
Overexpression of β-catenin reverses the effect of MICALL2 knockdown on CRC.** A** Western blotting was used to examine the expression of MICALL2 and β-catenin in the MICALL2-silenced HCT8 and control cell line transfected with β-catenin-expressing plasmids. GAPDH was used as an internal control. **B** Growth curves of MICALL2-silenced HCT8 and control cell line transfected with β-catenin-expressing plasmids. **C** Representative images (left) and quantification of wound area (right) of gap closure of MICALL2-silenced HCT8 and control cell line transfected with β-catenin-expressing plasmids, respectively. **D** A working model for MICALL2 functions in colorectal cancer cells. In the normal colonic epithelial cells, TRIM21 binds to MICALL2 and promotes the ubiquitination and degradation of MICALL2, thereby suppressing the growth and migration of normal colonic epithelial cells. However, in colorectal cancer cells, reduced TRIM21 expression leads to decreased ubiquitination and degradation of MICALL2, as a result, the up-regulated MICALL2 promotes the tumorigenesis of colorectal cancer via the Wnt/β-catenin signaling pathway

To determine whether upregulation of β-catenin could attenuate the inhibitory effect of MICALL2 knockdown on CRC, MICALL2-stable knockdown HCT8 cell line and control cell line were transfected with β-catenin expressing plasmid or empty vector (Fig. 7A). As expected, the functional assays results indicated that the overexpression of β-catenin significantly reverses the tumorigenic growth and migration of HCT8 cell line with MICALL2-stable knockdown (Fig. 7B, C). Thus, these evidences indicate that MICALL2 knockdown inhibits the tumorigenesis of CRC through Wnt/β-catenin signaling pathway.

## Discussion

In this study, we found that the expression level of MICALL2 is up-regulated in human CRC tissues when compared with matched non-tumor tissues, and that high expression of MICALL2 is associated with poor prognosis of CRC patients. In colorectal cancer cells, TRIM21 interacts with MICALL2 and down-regulates it synergistically via ubiquitination and degradation, and negatively regulates the activities of MICALL2 in CRC. And, in vitro and in vivo studies demonstrated that MICALL2 promoted tumor cell growth and migration via activating Wnt/β-catenin signaling pathway. Taken together, we highlighted the vital role of MICALL2, as an E3 ubiquitinase TRIM21 substrate, in colorectal cancer progression by activating Wnt/β-catenin signaling pathway.

TRIM21, as a member of TRIM family, is composed of RING domain with E3 ubiquitin ligase activity, a B-box domain, a coiled-coil domain, PRY and SPRY domains at the C-terminus [30]. Currently, TRIM21 acts as tumor enhancer or suppressor depending on the cancer context by adversely influencing the crucial molecules in cancer progression, such as p53, Oct-1, Par-4, SALL4, SALL1, c-FLIP, BCL2, NF-κB, et al. [27]. For the first time, MICALL2 was fully characterized as a novel substrate of TRIM21 E3 ligase by demonstrating that (i) MICALL2 binds to TRIM21 in a PRY-SPRY domain–dependent manner; (ii) TRIM21 promote MICALL2 ubiquitylation and TRIM21–mediated MICALL2 degradation can be rescued by a proteasome inhibitor; (iii) TRIM21 shortens half-life of MICALL2 protein. Therefore, MICALL2 has been added to the growing list of TRIM21 substrates. At the beginning of this study, the increased MICALL2 mRNA and protein levels were discovered in human CRC tissues compared with levels in the control tissues. And, in colorectal cancer tissues, there is a negative correlation of MICALL2 expression level with TRIM21, which promotes the ubiquitination and degradation of MICALL2. Moreover, downregulation of TRIM21 was discovered in CRC and inhibited intestinal epithelial carcinogenesis [28]. These evidences could partially explain why MICALL2 protein levels are increased in CRC. However, it’s widely known that protein ubiquitination, an important post-translational modification, regulates the degradation of proteins, but not alteration of mRNA [31]. To fully characterize desregulation of MICALL2 in CRC, more transcriptomic data would be needed.

MICALL2 has specific structural properties that distinguish it from its family members and confers a variety of biological roles, including controlling cytoskeleton assembly, tight junction formation, and endosome trafficking [12, 13, 32, 33]. The bMERB domain in MICALL2 is a protein–protein interaction domain that could interact with Rab proteins [34], in the present study, bMERB domain is essential for MICALL2 to bind to TRIM21. As the majority of MICAL family members share the conserved bMERB domain, TRIM21 could therefore potentially bind and regulate other family members of MICAL. Recently, studies have also gradually converged on its role in carcinogenesis. For example, MICALL2-targeting-shRNA dramatically suppressed the proliferation, migration and invasion of ovarian cancer cells or gastric cancer cells [16, 35]. These results are in accordance with another published study demonstrating that MICALL2 depletion decreased NSCLC cell proliferation [36]. When preparing this manuscript, we learned that another group identified the higher expression of MICALL2 in CRC [37], however, biological role in colorectal cancer (CRC) remain elusive. Our functional experiments revealed that MICALL2 overexpression promotes the growth and migration of CRC, whereas knockdown of MICALL2 had the opposite impact. Thus, this is the first comprehensive study elucidating the functions of MICALL2 in CRC, which indicates that MICALL2 is regarded as a prospective molecular target for CRC therapy. Furthermore, TRIM21 knockdown partially reversed the cancer-promoting effects of MICALL2 in CRC cells, suggesting that TRIM21 may be a crucial regulatory molecule for MICALL-mediated oncogenic signaling in CRC.

Colorectal cancer is characterized by the aberrant activation of canonical Wnt/β-catenin signaling pathway [38]. Nuclear translocation of β-catenin, which initiates transcription of downstream genes, such as c-Myc, cyclin D1, and c-JUN, is a key component of this signaling pathway activation [39]. In this study, MICALL2 overexpression increases the total cellular and nuclear levels of β-catenin, and promotes the transcription of downstream molecules, c-Myc and cylcin D1. These findings are consistent with previous research indicating MICALL2 promotes the Wnt/β-catenin signaling pathway in ovarian cancer [16, 35]. β-catenin destruction complex is the core of the canonical Wnt signaling pathway by promoting the phosphorylation and ubiquitination of β-catenin to maintain the low level of cytosolic and nuclear β-catenin [40]. Our RNAseq analyses indicated that MICALL2 is related with “β-catenin destruction complex disassembly” geneset (Fig. 3B), suggesting future research directions to clarify the molecular mechanism of the MICALL2-activated Wnt/β-catenin signaling pathway.

In summary, we report that MICALL2 functions as an oncogene in the proliferation and migration of CRC cells in vitro and in vivo. Mechanistically, MICALL2 as substrates of E3 ligase TRIM21 activates the Wnt/β-catenin signaling pathway in CRC (Fig. 7D). Our findings contribute to the understanding of the underlying role and molecular mechanisms of MICALL2 in the tumorigenesis and progression of CRC. Thus, this study suggested that targeted disruption of MICALL2 might be a therapeutic approach for CRC.

## Supplementary Information

**Additional file 1.** Supplementary Figure.

**Additional file 2.** Supplementary Table.

## Data Availability

The datasets used and/or analysed during the current study are available from the corresponding author on reasonable request.
