# Supplementary material for: MICALL2 as a substrate of ubiquitinase TRIM21 regulates tumorigenesis of colorectal cancer
Source: Cell Commun Signal. 2022 Oct 28;20:170. doi: 10.1186/s12964-022-00984-3 (PMC9615392; doi:10.1186/s12964-022-00984-3)
Supplement: Supplementary file 2 — Additional file 1. Supplementary Figure. [file 12964_2022_984_MOESM2_ESM.docx]

**Supplemental Figures**

**Figure S1**


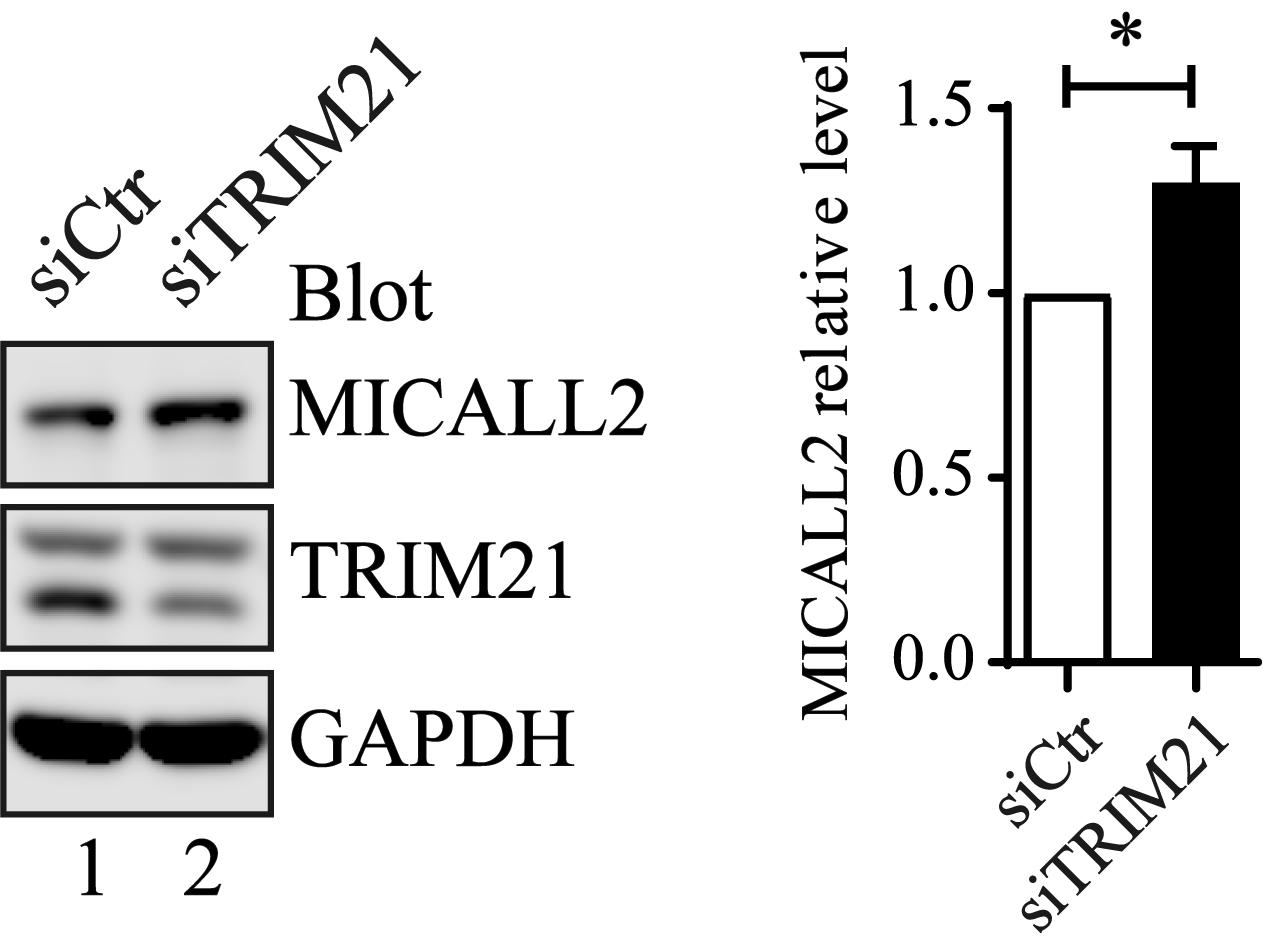


**Figure S1 The effect of TRIM21 knockdown on the MICALL2 protein level in CRC.** The expression of MICALL2 in the TRIM21-silenced HCT8 cell line (left) were measured by Western blotting analysis, and quantification of MICALL2 protein levels in different groups were compared to those of GAPDH (right).
