# Supplementary material for: MICALL2 as a substrate of ubiquitinase TRIM21 regulates tumorigenesis of colorectal cancer
Source: Cell Commun Signal. 2022 Oct 28;20:170. doi: 10.1186/s12964-022-00984-3 (PMC9615392; doi:10.1186/s12964-022-00984-3)
Supplement: Supplementary file 3 — Additional file 2. Supplementary Table. [file 12964_2022_984_MOESM3_ESM.docx]

**Table S1 Proteins identified by immunoprecipitation with the MICALL2 antibody followed by Mass Spectrometry analysis**

| **Accession** | **Gene names** | **Coverage (%)** | **Descriptions** | **# Unique Peptides** | **Protein score** |
| --- | --- | --- | --- | --- | --- |
| P19474 | TRIM21 | 37.47 | E3 ubiquitin-protein ligase TRIM21 | 16 | 733.36 |
| P68371 | TUBB4B | 37.75 | Tubulin beta-4B chain | 3 | 500.53 |
| Q12904 | AIMP1 | 44.23 | Aminoacyl tRNA synthase complex-interacting multifunctional protein 1 | 11 | 441.53 |
| P07437 | TUBB | 31.08 | Tubulin beta chain | 1 | 436.92 |
| P68363 | TUBA1B | 29.27 | Tubulin alpha-1B chain | 11 | 360.49 |
| P67809 | YBX1 | 32.10 | Y-box-binding protein 1 | 6 | 358.17 |
| P60709 | ACTB | 33.07 | Actin, cytoplasmic 1 | 11 | 354.13 |
| Q02878 | RPL6 | 39.24 | 60S ribosomal protein L6 | 10 | 308.20 |
| P22626 | HNRNPA2B1 | 28.90 | Heterogeneous nuclear ribonucleoproteins A2/B1 | 7 | 270.30 |
| P06748 | NPM1 | 30.27 | Nucleophosmin | 8 | 261.96 |
| P68104 | EEF1A1 | 21.43 | Elongation factor 1-alpha 1 | 7 | 251.60 |
| P07355 | ANXA2 | 27.14 | Annexin A2 | 9 | 246.76 |
| Q13155 | AIMP2 | 25.63 | Aminoacyl tRNA synthase complex-interacting multifunctional protein 2 | 6 | 219.22 |
| P62753 | RPS6 | 20.48 | 40S ribosomal protein S6 | 5 | 217.36 |
| Q13283 | G3BP1 | 30.90 | Ras GTPase-activating protein-binding protein 1 | 10 | 200.11 |
| P31146 | CORO1A | 19.96 | Coronin-1A | 9 | 197.74 |
| P62805 | H4C1 | 42.72 | Histone H4 | 4 | 197.05 |
| P81605 | DCD | 42.73 | Dermcidin | 5 | 193.43 |
| P62424 | RPL7A | 23.31 | 60S ribosomal protein L7a | 6 | 190.67 |
| P56470 | LGALS4 | 20.12 | Galectin-4 | 5 | 176.80 |
| Q8N257 | H2BU1 | 40.48 | Histone H2B type 3-B | 5 | 173.80 |
| P62701 | RPS4X | 22.05 | 40S ribosomal protein S4, X isoform | 7 | 160.01 |
| P18124 | RPL7 | 18.55 | 60S ribosomal protein L7 | 5 | 155.75 |
| P62917 | RPL8 | 13.23 | 60S ribosomal protein L8 | 3 | 151.04 |
| P26373 | RPL13 | 27.49 | 60S ribosomal protein L13 | 6 | 145.50 |
| O14757 | CHEK1 | 11.76 | Serine/threonine-protein kinase Chk1 | 5 | 145.19 |
| P61313 | RPL15 | 26.47 | 60S ribosomal protein L15 | 6 | 143.56 |
| Q12905 | ILF2 | 13.33 | Interleukin enhancer-binding factor 2 | 4 | 142.29 |
| P36578 | RPL4 | 16.86 | 60S ribosomal protein L4 | 6 | 140.81 |
| P51991 | HNRNPA3 | 12.70 | Heterogeneous nuclear ribonucleoprotein A3 | 3 | 140.57 |
| P35637 | FUS | 6.84 | RNA-binding protein FUS | 2 | 134.68 |
| P61978 | HNRNPK | 13.17 | Heterogeneous nuclear ribonucleoprotein K | 6 | 130.97 |
| P62241 | RPS8 | 28.37 | 40S ribosomal protein S8 | 5 | 127.81 |
| P61006 | RAB8A | 27.05 | Ras-related protein Rab-8A | 3 | 123.02 |
| P23396 | RPS3 | 26.75 | 40S ribosomal protein S3 | 6 | 121.90 |
| O00330 | PDHX | 8.38 | Pyruvate dehydrogenase protein X component, mitochondrial | 4 | 119.96 |
| Q8NC51 | SERBP1 | 9.31 | Plasminogen activator inhibitor 1 RNA-binding protein | 3 | 116.96 |
| P15880 | RPS2 | 18.43 | 40S ribosomal protein S2 | 5 | 116.73 |
| P84098 | RPL19 | 13.27 | 60S ribosomal protein L19 | 2 | 114.71 |
| Q07020 | RPL18 | 21.81 | 60S ribosomal protein L18 | 4 | 108.75 |
| P62851 | RPS25 | 15.20 | 40S ribosomal protein S25 | 2 | 104.62 |
| Q9UJZ1 | STOML2 | 8.99 | Stomatin-like protein 2, mitochondrial | 2 | 102.88 |
| P08559 | PDHA1 | 7.95 | Pyruvate dehydrogenase E1 component subunit alpha, somatic form, mitochondrial | 3 | 101.08 |
| P16403 | H1-2 | 26.76 | Histone H1.2 | 4 | 99.40 |
| P39023 | RPL3 | 13.90 | 60S ribosomal protein L3 | 5 | 94.91 |
| P50914 | RPL14 | 12.09 | 60S ribosomal protein L14 | 3 | 92.78 |
| P62269 | RPS18 | 19.08 | 40S ribosomal protein S18 | 3 | 90.45 |
| P62277 | RPS13 | 19.21 | 40S ribosomal protein S13 | 3 | 88.03 |
| P36957 | DLST | 5.52 | Dihydrolipoyllysine-residue succinyltransferase component of  2-oxoglutarate dehydrogenase complex, mitochondrial | 2 | 86.34 |
| P50552 | VASP | 3.16 | Vasodilator-stimulated phosphoprotein | 1 | 85.72 |
| P62979 | RPS27A | 16.03 | Ubiquitin-40S ribosomal protein S27a | 2 | 85.58 |
| Q8TB37 | NUBPL | 14.42 | Iron-sulfur protein NUBPL | 5 | 82.37 |
| P04908 | H2AC4 | 21.54 | Histone H2A type 1-B/E | 2 | 80.05 |
| P07910 | HNRNPC | 13.07 | Heterogeneous nuclear ribonucleoproteins C1/C2 | 4 | 78.40 |
| P46781 | RPS9 | 18.56 | 40S ribosomal protein S9 | 4 | 72.23 |
| Q9Y3Q8 | TSC22D4 | 5.82 | TSC22 domain family protein 4 | 2 | 71.65 |
| P05062 | ALDOB | 7.42 | Fructose-bisphosphate aldolase B | 1 | 71.10 |
| O75367 | MACROH2A1 | 5.38 | Core histone macro-H2A.1 | 1 | 70.82 |
| P47914 | RPL29 | 9.43 | 60S ribosomal protein L29 | 1 | 70.16 |
| P0DOX5 | --- | 3.34 | Immunoglobulin gamma-1 heavy chain | 2 | 67.64 |
| P05141 | SLC25A5 | 7.38 | ADP/ATP translocase 2 | 2 | 61.99 |
| P04899 | GNAI2 | 4.23 | Guanine nucleotide-binding protein G(i) subunit alpha-2 | 1 | 61.21 |
| P05388 | RPLP0 | 6.94 | 60S acidic ribosomal protein P0 | 2 | 57.85 |
| P62280 | RPS11 | 14.56 | 40S ribosomal protein S11 | 3 | 57.36 |
| P16401 | H1-5 | 8.85 | Histone H1.5 | 1 | 56.34 |
| P27635 | RPL10 | 8.88 | 60S ribosomal protein L10 | 2 | 56.34 |
| P46779 | RPL28 | 16.06 | 60S ribosomal protein L28 | 2 | 54.52 |
| P62829 | RPL23 | 10.71 | 60S ribosomal protein L23 | 1 | 53.81 |
| P31943 | HNRNPH1 | 7.13 | Heterogeneous nuclear ribonucleoprotein H | 3 | 52.38 |
| P61254 | RPL26 | 11.03 | 60S ribosomal protein L26 | 2 | 51.36 |
| P68431 | H3C1 | 11.76 | Histone H3.1 | 2 | 50.53 |
| P61353 | RPL27 | 6.62 | 60S ribosomal protein L27 | 1 | 49.19 |
| P11177 | PDHB | 5.29 | Pyruvate dehydrogenase E1 component subunit beta, mitochondrial | 2 | 48.35 |
| P83731 | RPL24 | 10.83 | 60S ribosomal protein L24 | 2 | 47.58 |
| P62140 | PPP1CB | 3.06 | Serine/threonine-protein phosphatase PP1-beta catalytic subunit | 1 | 47.37 |
| P30050 | RPL12 | 5.45 | 60S ribosomal protein L12 | 1 | 46.85 |
| P49207 | RPL34 | 20.51 | 60S ribosomal protein L34 | 3 | 45.74 |
| P02042 | HBD | 6.80 | Hemoglobin subunit delta | 1 | 45.74 |
| P26368 | U2AF2 | 3.79 | Splicing factor U2AF 65 kDa subunit | 2 | 45.39 |
| Q9Y383 | LUC7L2 | 6.63 | Putative RNA-binding protein Luc7-like 2 | 2 | 45.32 |
| P62249 | RPS16 | 6.85 | 40S ribosomal protein S16 | 1 | 45.23 |
| P0DN76 | U2AF1L5 | 5.42 | Splicing factor U2AF 35 kDa subunit-like protein | 1 | 45.20 |
| Q01105 | SET | 3.79 | Protein SET | 1 | 43.14 |
| Q02543 | RPL18A | 5.68 | 60S ribosomal protein L18a | 1 | 42.57 |
| Q8TDB8 | SLC2A14 | 1.15 | Solute carrier family 2, facilitated glucose transporter member 14 | 1 | 41.21 |
| P46777 | RPL5 | 6.73 | 60S ribosomal protein L5 | 3 | 40.88 |
| P40429 | RPL13A | 8.37 | 60S ribosomal protein L13a | 2 | 40.17 |
| P62906 | RPL10A | 13.82 | 60S ribosomal protein L10a | 3 | 39.97 |
| Q99623 | PHB2 | 10.03 | Prohibitin-2 | 2 | 39.66 |
| P18621 | RPL17 | 12.50 | 60S ribosomal protein L17 | 2 | 39.54 |
| P62854 | RPS26 | 7.83 | 40S ribosomal protein S26 | 1 | 39.11 |
| Q08170 | SRSF4 | 1.82 | Serine/arginine-rich splicing factor 4 | 1 | 38.21 |
| P61247 | RPS3A | 3.41 | 40S ribosomal protein S3a | 1 | 37.81 |
| P62263 | RPS14 | 7.28 | 40S ribosomal protein S14 | 1 | 37.09 |
| A0A0C4DH72 | IGKV1-6 | 13.68 | Immunoglobulin kappa variable 1-6 | 1 | 37.01 |
| P51153 | RAB13 | 13.79 | Ras-related protein Rab-13 | 1 | 36.89 |
| P06733 | ENO1 | 2.07 | Alpha-enolase | 1 | 36.70 |
| P84103 | SRSF3 | 14.02 | Serine/arginine-rich splicing factor 3 | 2 | 36.65 |
| Q07666 | KHDRBS1 | 7.67 | KH domain-containing, RNA-binding, signal transduction-associated protein 1 | 2 | 36.34 |
| Q15233 | NONO | 2.97 | Non-POU domain-containing octamer-binding protein | 1 | 36.08 |
| Q6PID8 | KLHDC10 | 1.58 | Kelch domain-containing protein 10 | 1 | 35.56 |
| Q00325 | SLC25A3 | 3.31 | Phosphate carrier protein, mitochondrial | 1 | 34.30 |
| P62750 | RPL23A | 7.05 | 60S ribosomal protein L23a | 1 | 33.78 |
| Q13190 | STX5 | 2.82 | Syntaxin-5 | 1 | 33.56 |
| P27169 | PON1 | 3.10 | Serum paraoxonase/arylesterase 1 | 1 | 32.58 |
| O75477 | ERLIN1 | 3.45 | Erlin-1 | 1 | 32.23 |
| P31025 | LCN1 | 4.55 | Lipocalin-1 | 1 | 31.62 |
| P32322 | PYCR1 | 3.76 | Pyrroline-5-carboxylate reductase 1, mitochondrial | 1 | 30.87 |
| P62266 | RPS23 | 7.69 | 40S ribosomal protein S23 | 1 | 30.79 |
| P62081 | RPS7 | 15.46 | 40S ribosomal protein S7 | 2 | 30.70 |
| Q9NZT1 | CALML5 | 5.48 | Calmodulin-like protein 5 | 1 | 30.61 |
| Q8ND56 | LSM14A | 2.16 | Protein LSM14 homolog A | 1 | 29.09 |
| P46776 | RPL27A | 7.43 | 60S ribosomal protein L27a | 1 | 28.55 |
| P09622 | DLD | 2.16 | Dihydrolipoyl dehydrogenase, mitochondrial | 1 | 25.67 |
| P42766 | RPL35 | 8.13 | 60S ribosomal protein L35 | 1 | 23.30 |
| Q9Y6H3 | ATP23 | 2.44 | Mitochondrial inner membrane protease ATP23 homolog | 1 | 22.82 |
| P04406 | GAPDH | 2.99 | Glyceraldehyde-3-phosphate dehydrogenase | 1 | 22.01 |
| A0A087WW87 | IGKV2-40 | 5.79 | Immunoglobulin kappa variable 2-40 | 1 | 21.48 |
